# Research on key acoustic characteristics of soundscapes of the classical Chinese gardens

**DOI:** 10.1038/s41598-023-39457-z

**Published:** 2023-08-03

**Authors:** Wei Chen, Juanjuan Liu

**Affiliations:** 1 College of Landscape Architecture Horticulture, Southwest Forestry University, Kunming, 650224 China; 2 College of Landscape Architecture Horticulture, Southwest Forestry University, Kunming, 650224 China

**Keywords:** Environmental sciences, Environmental impact

## Abstract

Soundscapes have played an important role in the design and building of classical Chinese gardens. In Chinese classical poetry, biophonies such as bird calls, and geophonies such as wind, are the preferable sound sources. Although these major sound sources have been categorized and summarized by scholars extensively, little research has been conducted to analyze the physical characteristics and preference matrix of these preferred sound sources. Moreover, the perceived loudness of sound in classical Chinese gardens has received more attention from scholars than acoustic frequency. In this study, we selected 12 sound sources that are most typically present in classical Chinese gardens based on extensive literature research on Chinese classical poetry, and acquired respective audio samples from the BBC’s library of Sound Effects. Through the spectrogram analyses, pitch detection algorithm and LSTM audio classification methods, the sound sources were classified into discrete sound sources with pitch variation and continuous sound sources with spectral characteristics of white noise or pink noise. The reasoning behind the preference for these two types of sound sources was then discussed from physical and mental healing perspectives, which aims to help provide perspectives on the associated implications in the planning of urban green spaces.

## Introduction

The term "soundscape" was first coined by Murray Schafer in his book "The tuning of the world" published in 1997^1,2^. In 2014, the International Organization for Standardization systematically elaborated the definition of the soundscape: "acoustic environment as perceived or experienced and/or understood by a person or people, in context"^3^. It also defined the constituent elements of the soundscape as sound elements, environmental elements and audio receivers. The physical characteristics of sound include loudness, pitch, and timbre. In the discipline of soundscape ecology, sound is classified into three distinct types: biophonies, geophonies and anthrophonies^4,5^.

Classical Chinese gardens reflect the profound metaphysical beauty of Chinese culture in scrupulous garden design, and they are a significant component of world cultural heritage. The creation of classical Chinese gardens places a strong emphasis on crafting a multisensorial experience through the sensescape, in which soundscape plays a critical role^6^. Early studies have revealed a remarkable consistency in the adoption of similar soundscapes and sound sources in classical Chinese gardens^7^. However, most of these studies provided only a summary and categorization, without further analysing the physical characteristics of these sound sources and the reasoning behind the preference matrix.

Most of the soundscape research focuses on loudness, despite the fact that physical characteristics of sound also include frequency, timbre, and duration. Scholars have pointed out that once the loudness of sound remains within people’s comfort zone, evaluation of soundscape will mainly depend on the type of sound source and personal subjective preference, while ignoring the influence of sound frequency, timbre and other physical properties on preference^8,9^. Despite the profound impact of frequency attributes on human sound perception and the growing recognition of their therapeutic effects on physical and mental well-being^10^, relevant research in this domain remains relatively sparse. Many scholars use soundscape data, combined with theories and methods of sociology, psychology or physiology to evaluate soundscape. On the one hand, such as Hunte and Jo, etc. concluded that the sound of water, wind, birds and other natural sounds have healing effects on human beings, but did not explore the healing mechanism of these soundscapes in depth; on the other hand^11,12^, Casc detailed standardizes the standard of using the frequency characteristics of sound to measure species diversity, and discusses the role of sound frequency in the study of species diversity^13^. This study takes the most recorded 12 soundscape in classical Chinese gardens as the research object, analyzes its frequency characteristics, discusses its healing mechanism, and provides a frequency perspective for the study of soundscape healing mechanism. Audio data for these identified soundscapes is sourced from the BBC Sound Effects website, allowing for an acoustic analysis that focuses solely on the frequency dimension, while mitigating the influence of the physical variable of loudness. By analyzing sound spectrograms, Bai et al.^14^ divided sound into two types based on sound duration: discrete and continuous. Discrete sounds tend to be associated with musical melodies; a melody is composed of two or more tones, which are discrete vocal events with pitch^15^. Continuous sound, on the other hand, forms a spectrogram through methods such as Fourier transform^16^. The spectrogram is the basis for distinguishing between colored noise and white noise^17^. Not all noises are harmful to human health. Pink noise and white noise can provide a soothing quality, by masking out disturbing sounds from the external environment to inhibit the activation of brain activity, which is evident from the reduced complexity of electroencephalogram (EEG) recordings^18^.

The ancient Chinese culture attached great importance to sound perception. In ancient Chinese idioms, when the words "ear" and "eye" are mentioned together in an idiom, the word "ear" is always placed before the word "eye"; the sound comes first, and the form follows. Soundscapes have also been recorded in various ancient texts. Wu et al.^6^ have conducted an extensive literature review on the soundscape present in the "Book of Songs" (also known as “Shijing”), and found that 86 of the 305 poems in the "Book of Songs" involve soundscapes.

In classical Chinese gardens, the creation and crafting of soundscapes is a meticulous and significant art form with a longstanding history. Tang et al.^19^ have conducted an extensive literature review on the soundscape present in "Yuanye", the first comprehensive garden art monograph in China. They found 21 descriptions of soundscapes, with biophonies (e.g. bird calls) as the most commonly recorded, followed by geophonies (e.g. water and wind sounds), and lastly anthrophonies (e.g. singing and musical performance). Xie and Ge^7^ also conducted a literature review on the important contemporary works of classical Chinese gardens, and found a similar ranking of the soundscapes based on their frequency of occurrence^20–25^.

**Figure 1:**
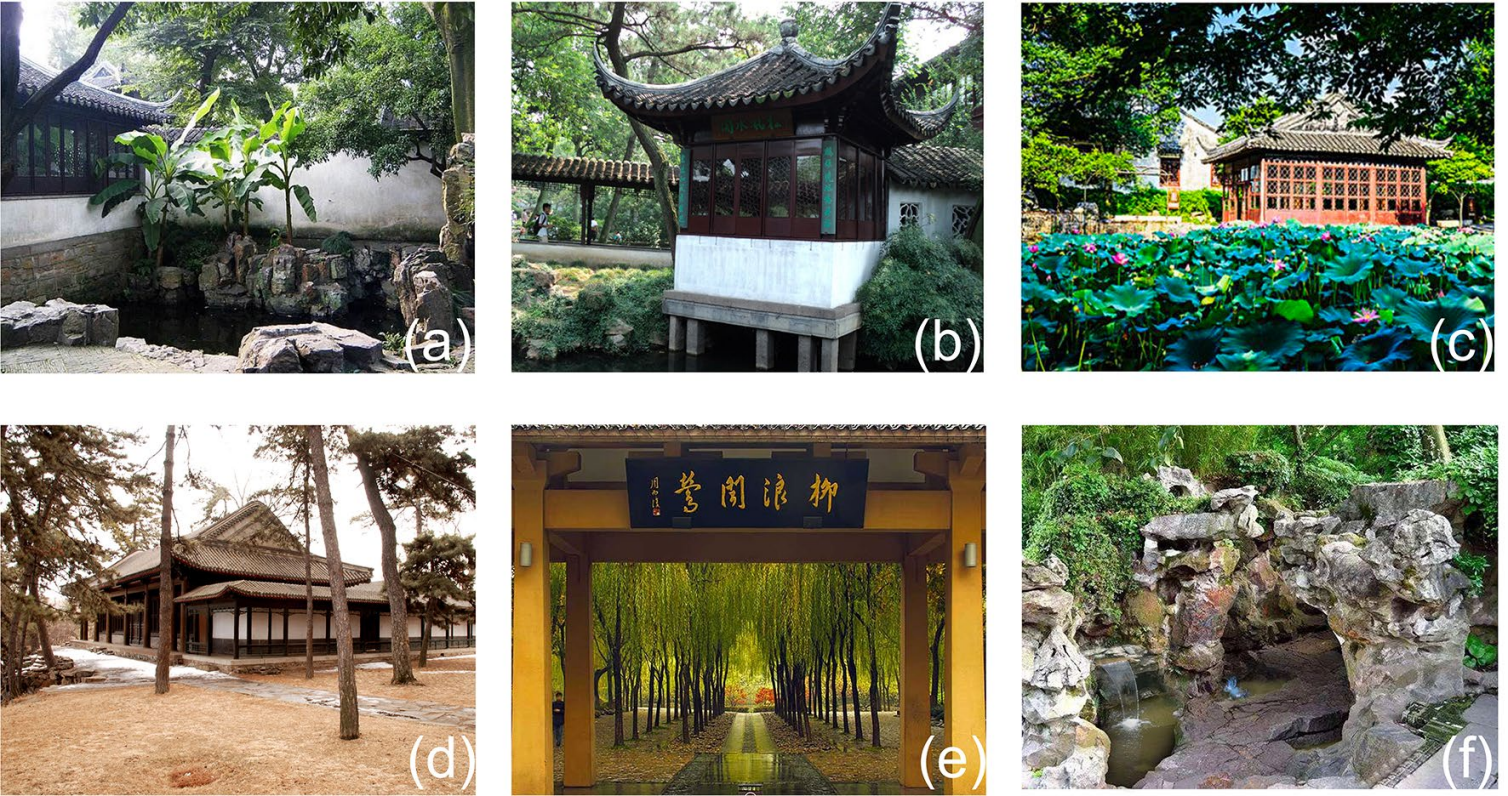
Site photos of existing classical Chinese gardens with distinct soundscapes. Inset images show soundscape representatives of the 6 in existing classical Chinese gardens.From left to right: (**a**) Tingyuxuan; (**b**) Songfengting; (**c**) Liutingge; (**d**) Wanhesongfengdian; (**e**) Liulangwenying; (**f**) Bayinjian.

Although the ancients did not systematically acquire relevant acoustic theory and technology, their understanding and experiences have been demonstrated in the mastery of soundscapes in the making of the garden landscape^20,23^. There are many well-known soundscapes in existing classical Chinese gardens. In the Humble Administrator’s Garden, the "Pine Wind Pavilion" (Songfengting) has a horizontal banner inscribed with the phrase "Listen to the Pine Wind", which is an extract from the poem "I love the pine wind, and the courtyard is planted with pine trees. Every time I hear the sound, I am delighted." Fig. 1a "Rain Pavilion" (Tingyuxuan) has a pond filled with lotus flowers in the front, and with Musa trees and bamboo planted by the side. When raindrops fall on these different plants, they produce responses in listeners; often, these responses will be highly individual (i.e. differ between people) (Fig. 1b and c). Chengde Mountain Resort has a building dedicated to listening to the wind blowing through an old-growth pine forest (Fig. 1d). The "Orioles Singing in the Willow" (Liulangwenying) is a lakefront park located on the southeast bank of the West Lake, which is famous for bird calls from orioles (Fig. 1e). The “Octave Stream” (Bayinjian) in Jichang Garden demonstrates skilful stonework, creating an enclosed environment which isolates noise from the outside world and amplifies the trickling sound of spring water (Fig. 1f).Soundscape research centers on two major aspects: the objective assessment of the physical characteristics of sound, and the subjective evaluation of the perception of sound. However, most research on soundscape perception focused mainly on the perceived loudness, despite the fact that frequency and timbre are equally important components of the physical characteristics of sound^26^. In this paper, we conducted an extensive systematic literature review of related soundscape research and selected 12 sound sources that are most typically present in classical Chinese gardens. We then collected the respective audio samples from the BBC’s library of Sound Effects to classify these 12 audio samples into discrete and continuous sounds based on spectrogram analyses. Their frequency distribution was further analyzed by the Pitch Estimation Algorithm and LSTM neural network noise type judgment method based on the theories of musical tones, melody and colored noise classification. According to the results, the frequency distribution characteristics of discrete sounds indicate a pitch change, while continuous sounds show white noise or pink noise. The preference mechanism for these sound sources from the perspective of healing and health benefits will be discussed in this paper. This study presents a novel approach by explaining the physical attributes and preference mechanisms of soundscapes in classical gardens from the perspective of sound frequency. The innovation of this study can be observed in two aspects.

Firstly, methodological innovation: The utilization of LSTM neural networks and principles from music theory to analyze audio data led to the discovery of frequency-based features that are favored by individuals. Specifically, it was found that the frequency distribution of continuous sound sources conforms to the distribution patterns of white noise and pink noise. This approach provides a unique methodology for understanding and interpreting the frequency characteristics of preferred sounds, that is discrete sounds exhibit two or more variations in pitch. Secondly, theoretical innovation: The study offers an explanation from a therapeutic perspective as to why people are drawn to sounds with these frequency features. By highlighting the potential healing effects of these sounds, the research contributes to the improvement of urban green spaces and the enhancement of residents’ physical and mental well-being. The study provides a frequency-based perspective and serves as a valuable reference for the enhancement of acoustic environments in urban green spaces and the promotion of residents’ overall health.

## Methods

### Research sample selection

In a study by Xie and Ge^7^ the soundscapes in six important contemporary historical books about traditional Chinese gardens were carefully examined: History of Chinese Classical Gardens^27^, History of Ancient Chinese Gardens^28,29^, Lingnan Garden Art^30^, and History and Culture of Xishu Memorial Gardens^31^, Bashu Garden Art^32^, and Xishu Garden^33^. The sound source types were classified into 50 non-biophonies and 12 biophonies. The detailed classification can be found in Supplemental File 1. In this study, based on the compilation results by Xie and considering both the existing soundscapes found in gardens and the soundscapes recorded in ancient Chinese poetry, we have selected 12 of the most common and representative soundscape types as the focus of our research. They are: (1) rain in a bamboo forest, (2) rain on Musa trees, (3) rain on a lotus leaf, (4) wind blowing through a pine forest, (5) wind blowing through a bamboo forest, (6) small stream, (7) waterfall, (8) spring, (9) the common cuckoo calls, (10) the Indian cuckoo calls, (11) the red-crowned crane calls, and (12) oriole calls. The 12 sound types selected in this paper were classified based on soundscape ecology as shown in Table 1.

**Table 1:** Classification of sample sounds.

The type of sound source		Sound Sources sample
Biophonies	Bird calls	The common cuckoo, Indian cuckoo, red-crowned crane, and oriole (Chengde Mountain Resort, Gen Yue, Kaifeng, Jingyi Park, Beijing)
Non-biophonies	Wind	Wind blowing through the pine or bamboo forest (Humble Administrator’s Garden, Suzhou)
	Pitter-patter	Rain sounds (on Musa leaves, bamboo forest, and lotus leaves) (Humble Administrator’s Garden, Suzhou, Jichang Park, Wuxi)
	Running water	Waterfalls, springs, and small streams (Garden of Eternal Spring (Changchunyuan), Beijing, Old Summer Palace/Yuanmingyuan Park, Beijing, Shuqing Garden, Xiyuan, Beijing)

### The sound acquisition method

The audio data for the 12 identified soundscape categories in this study were exclusively sourced from the BBC Sound Effects, renowned as an authoritative sound archive website. Field recording within classical gardens was not employed to acquire the audio data for two primary reasons. Firstly, the sound waves generated by the same vibrating object at different locations propagate through the medium of air, resulting in variations in loudness within the recorded sound data, while the fundamental frequency remains consistent. Secondly, different locations often introduce distinct interfering noises, requiring meticulous processing, including track separation and noise reduction, to eliminate such disturbances and enable accurate frequency analysis. Dealing with interfering noises captured during on-site recordings poses even greater challenges. To address interfering noises, the following methods were employed for audio data processing. Firstly, to eliminate incidental sound interferences occurring within specific time intervals, Matlab 2021 software was utilized to automatically extract sound clips from the audio materials. These clips, each spanning 1 min, were extracted at 10-min intervals, resulting in a total of 34 min of meticulously selected audio data. Secondly, to ensure the quality of the target sounds by removing any interfering elements, Cubase 8 software was employed for comprehensive noise reduction within the sound files. Lastly, Adobe Audition software was utilized to merge the processed audio tracks, facilitating streamlined analysis of the sound’s frequency characteristics in subsequent stages. The resulting processed sound data acquired through these procedures is deemed suitable for utilization within the scope of this research study.

### Acoustic analysis method

**Figure 2:**
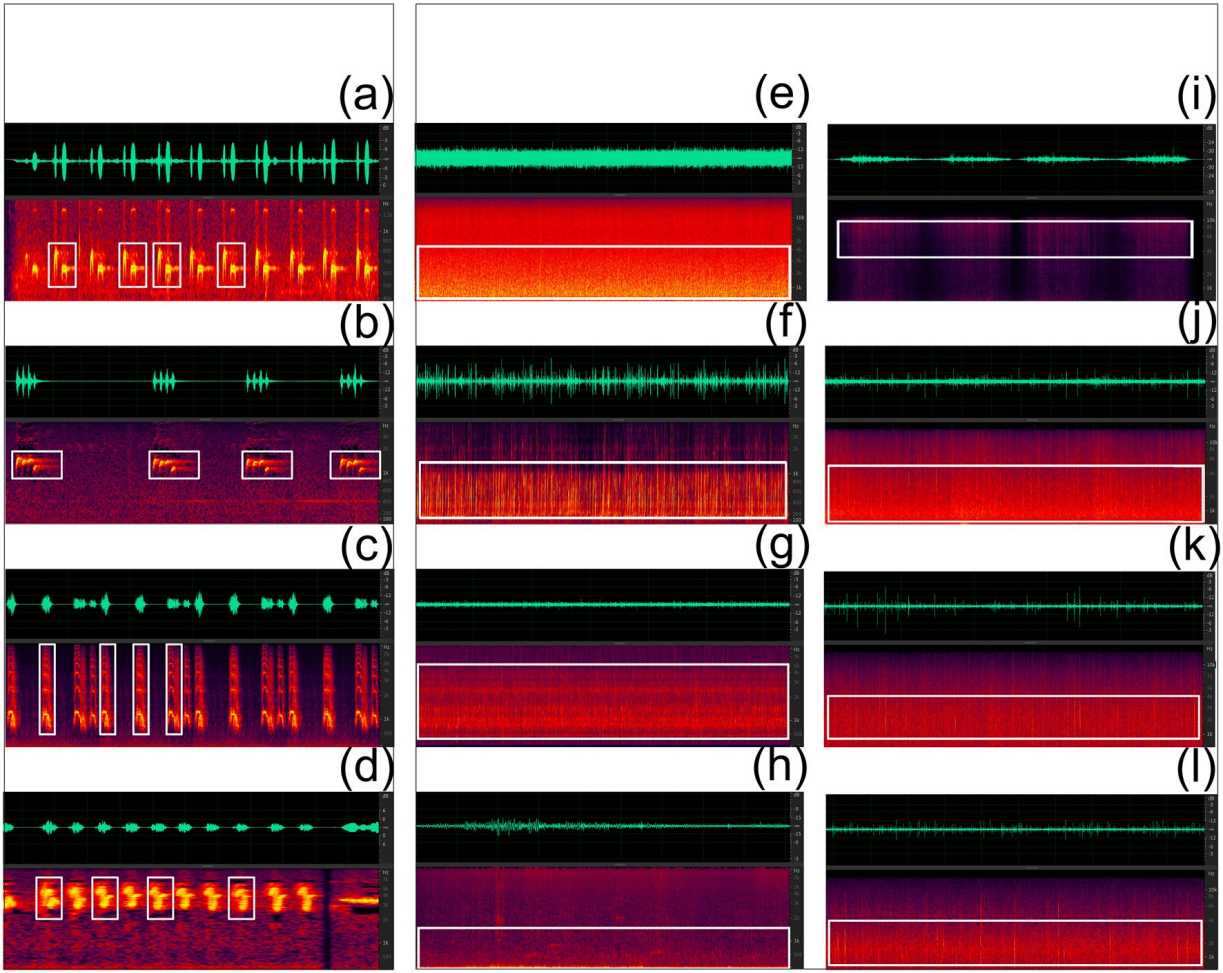
Sound spectrogram and time-domain diagram of the utterance event. Inset images with Discrete sounds on the left and Continuous sounds on the right. (**a**) The common cukoo; (**b**) Indian cukoo; (**c**) Red-crowned crane; (**d**) Oriole; (**e**) Waterfalls; (**f**) Springs; (**g**) Small streams; (**h**) Wind blowing through the bamboo forest; (**i**) Wind blowing through the pine forest; (**j**) Rain sounds on Musa leaves; (**k**) Rain sounds on bamboo forest; (**l**) Rain sounds on lotus leaves.

The sound spectrograph produces a visual record in the form of a spectrogram, which describes the distribution of energy in frequency and time^34^. The energy of the sound is visualized by the intensity of the color representing that sound, so louder sounds with more energy tend to have brighter, more intense colors^35^. An utterance event is defined as the duration between the appearance and ending of an acoustic signal. An example of a sound spectrogram and the time domain diagram of an utterance event is shown in Fig. 2.

The pitch estimation method can be divided into a single-tone pitch estimation and multi-tone pitch estimation. The sound samples selected in this study are all single-tone pitch estimations. Import the processed sample audio into Cubase 8 software, use the VariAudio plug-in, box the area with the most concentrated energy according to the result display, and compare the boxed area with the right pitch scale to get the sound pitch of the area with the most concentrated sound energy.

The noise signal is a relatively complex time-series signal with various types. It is difficult to classify noise by spectrum analysis, as the accuracy is overly dependent on the human experience.

Noise classification is achieved by converting noise into a series of data, and then performing digital long-sequence classification to obtain the type of noise. This paper uses the Long Short-Term Memory (LSTM)-based noise classification method, builds an LSTM neural network for noise classification based on MATLAB, optimizes the neural network structure and generates several different noise signal data through simulation methods. Among these, 70% of the noise sequences are used as the neural network training set, and 30% are used as the test set to train the neural network. Finally, the accuracy of the LSTM neural network is verified and analyzed by using the real known types of noise data, and finally, the intelligent identification of noise signal types based on the LSTM neural network is realized. The code used in this study can be found at https://github.com/17872999/Scientific-Reports/blob/main/Sound%20classification.

## Results

### Sample sound feature analysis

As shown in Table 2, the 12 sound source samples can be divided into two types: discrete and continuous. Four audio recordings were classified as discrete sounds, and 8 as continuous sounds.

**Table 2:** The type and description of sound sources.

Type	Description	Sound sources sample
Discrete	The time to complete a sound event is short and it does not occur continuously, but it can recur in groups	Bird calls: the common cuckoo, Indian cuckoo, red-crowned crane, and oriole
Continuous	A sound event occurs continuously, but does not occur in groups	Waterfalls, springs, small streams; the wind blowing through a pine or bamboo forest; the rain on *Musa* leaves, pine forest, bamboo forest, or lotus leaves

### Discrete sound source has pitch variation

**Figure 3:**
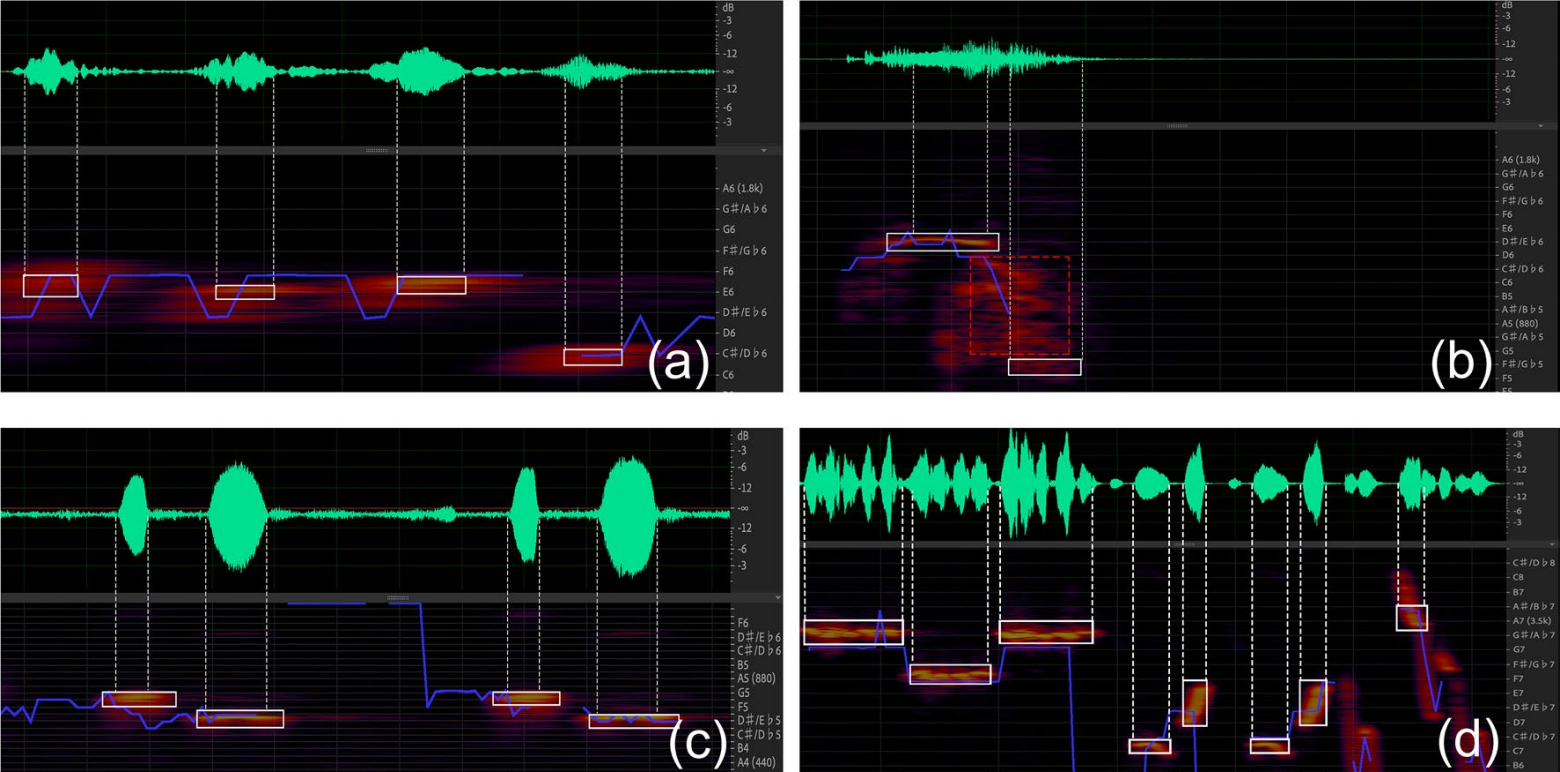
Pitch estimation results for each discrete sound sample. (**a**) Pitch changes in the Indian cuckoo bird call; (**b**) pitch changes in the red-crowned call; (**c**) pitch changes in the common cuckoo bird call; (**d**) pitch changes in the oriole bird call.

By using the frequency analysis marker tool in Adobe Audition CC 2019 software to perform spectral frequency analysis on the four discrete sound sources, we found that the single vocalization of Indian cuckoo, red-crowned crane, common cuckoo and oriole all have two or more pitch changes (Table 3). The order of pitch changes in the Indian cuckoo bird call is ^#^f^3^-^♮^f^3^-^♮^f^3^-d^3^, f^3^-e^3^-e^3^-^#^c^3^ (Fig. 3a); in the red-crowned crane bird call: ^b^e^3^ ↘ ^b^g^2^ (Fig. 3b); in the common cuckoo bird call: ^#^f^2^-^#^*d*^*2*^ (Fig. 3c); and in the oriole bird call: ^#^g^4^- f^4^- ^#^g^4^; e^4^- ^#^c^4^- ^#^d^4^- e^4^- ^#^c^4^- ^#^d^4^ (Fig. 3d).

**Table 3:** Tones contained in discrete sample sounds.

The name of the acoustic source	The name of the pitch				
Indian cuckoo	^#^c^3^	e^3^	^♮^f^3^	^#^f^3^	
Red-crowned crane	^b^e^3^	^b^g^2^			
Common cuckoo	^#^d^2^	^#^f^2^			
Oriole	^#^c^4^	^#^d^4^	e^4^	f^4^	^#^g^4^

### The classification of continuous sound sources

Common colored noises include pink noise, white noise, red noise, orange noise, blue noise, purple noise, gray noise, brown noise, and black noise. The power spectral density function of these noises is 1/f ^X^, where *X* is a measure of how fast the energy dissipates with frequency. The “color” of noise is determined based on the power spectral density^36–38^. All continuous sound sources analyzed in this study belong to white noise and pink noise. For white noise, its relative influence per unit frequency is 1 (i.e. *x* = 1), hence the distribution of the frequency component power of white noise in the audible range (0–20 kHz) is uniform. The human ear is more sensitive to high-frequency sounds, and white noise sounds like a rustling sound. The energy distribution of white noise is uniform, and the power spectral density has no relationship with frequency^39–41^. There are two white noise sources in the eight continuous sound samples selected in this study, namely the sound of small streams and the sound of wind blowing through the pine forest. Pink noise differs from white noise in its frequency component power being mainly concentrated in the middle to low frequency bands, so it is often considered to be a "pleasant" noise^14,42^. The energy gradually decays as the frequency increases, usually 3 dB per 1oct increase, which means that the octave energy distribution is uniformly equal^43^. The eight sound samples were classified as pink noise and white noise in Table 4.

**Table 4:** Colored noise classification for continuous sound samples.

White noise	Pink noise
Small streams	Waterfalls Springs Wind blowing through bamboo forest
Wind blowing through pine forest	Rain on *Musa leaves* Rain on bamboo forest rain on lotus leaves

## Discussion

Frequency-based sound sample types: According to our results, the studied audio samples can be divided into two sound types: discrete sound with pitch variation and continuous sound with frequency distribution characteristics of white noise or pink noise.

### The healing potential of different sound types

As a primary element of musical composition, the melody is defined as a succession of pitches in rhythm. Musical sounds possess three fundamental characteristics: volume, pitch, and timbre. Therefore, a sound composition must encompass these three elements. Firstly, it should generate audible sound. Secondly, it should comprise two or more musical tones with distinct pitches. Lastly, the individual tones should emanate from different vibrating objects, resulting in unique timbre, thereby forming a melody. In this study, four specific bird calls were selected based on their frequency and volume falling within the audible range and being recordable by sound receivers. Moreover, each bird call exhibits two or more noticeable variations in pitch. Finally, these bird calls originate from the vocal organs of birds, generating sounds through their unique patterns of vibration. Consequently, the four chosen bird calls in this study meet the necessary criteria for forming a melodic composition. We can conclude that discrete sound sources present in the soundscape of classical Chinese gardens all have melody characteristics.

Ball et al.^44^ proposed that the reason why people like hearing variation in musical tones is owing to the balance between surprise and predictability. From a psychological point of view, the human brain constantly relies on internal models to formulate predictions and make judgments on external obscure information. When it accurately predicts the plausible state of the outcome, the transmission and production of dopamine increase, producing ‘happiness’ feelings in a person.

The power spectral density of white noise is uniformly distributed in the entire frequency domain, and the energy carried by each frequency domain is basically the same^45^. The energy of pink noise is mainly concentrated in the middle to low frequency bands. According to the data analysis results, among the eight continuous sound samples in this study, six are pink noise and two are white noise. The sound that an infant experiences in a mother’s womb is considered to be white noise. Studies have shown that white noise has a significant effect on relieving people’s stress. White noise and pink noise are continuous monotonous sounds that suppress undesired sounds from the external environment in the form of resonance. Because of their soothing quality, white or pink noises are medically used to treat disorders such as mental distraction, tinnitus and insomnia^42,46,47^. We speculate that in the design and creation of soundscapes in classical Chinese gardens, continuous sound sources were intentionally selected due to their characteristics as white or pink noise, which have soothing and healing effects on humans.

Implications for contemporary soundscape design: Classical Chinese gardens demonstrate the mastery of using geophonies and biophonies to create pleasant soundscapes that are loved and appreciated by many. The sounds of wind, rain, water and bird calls re-create a sense of natural wilderness that heals body and mind. In general, people have an innate preference for natural sounds such as the rustling of leaves, the blowing of the wind, the flowing of water, and the songs of birds and insects. The frequencies of these sounds help relieve stress and provide health benefits. The contemporary urban acoustic environment is harsh, yet planners and designers pay far less attention to the soundscape of urban green spaces than designers of classical Chinese gardens. The design of soundscapes in contemporary urban green spaces should focus on the following three aspects:

1. Protect and create habitats for animals that use vocalization as a means of communication.

Ecological planning and design methods should be designed to attract more birds, insects, and other vocalizing animals so as to introduce biophonies that are favored by many people.

(2) Protect and create a natural environment that can provide high-quality geophonies.

Through the selection and configuration of planting palettes that facilitate wind and water sounds with the characteristics of white or pink noises, designers can mask out the undesired noises while creating a soothing acoustic environment for many to enjoy.

(3) Acknowledge the importance of sound frequencies in a soundscape.

Previous studies of soundscape preference mechanisms tended to focus on loudness, while rarely involving the frequency, despite the fact that sound frequency greatly affects human perception of sound. By studying people’s preference for particular sound frequency characteristics, one may apply this understanding in design methods to avoid or attenuate uncomfortable frequencies in soundscape creation. Examples include bird calls that do not possess pitch variation, such as crows and seagulls, as well as rain sounds that do not meet the characteristics of pink noise or white noise, such as rain hitting tin, plastic, and similar materials.

(4) Optimizing adverse acoustic environments through the introduction of discrete and continuous sound sources.

In environments with poor acoustic conditions where it is not feasible to eliminate sources of noise, particularly in residential areas and parks adjacent to busy urban roads, introducing discrete sound sources with varying pitches can help mitigate the impact. Examples of such discrete sound sources include the calls of common cuckoos, orioles, and Indian cuckoos. These sounds attract people’s attention and divert their focus away from other noises. Additionally, incorporating continuous sound sources with characteristics of pink noise or white noise, such as fountains, waterfalls, bamboo groves, pine forests, lotus ponds, and banana plants, can be beneficial. These elements help create a more soothing environment, reducing the propagation of external noise and alleviating feelings of irritation and anxiety caused by excessive noise.

## Conclusion

The soundscape encompasses the interplay between sound, human beings, and the surrounding environment. Presently, the study of sound elements in soundscapes primarily focuses on the loudness while overlooking the frequency. However, this research highlights the immense potential of sound frequency in enhancing both the physical and psychological well-being of individuals and elevating overall satisfaction with the soundscape. Therefore, it is imperative to intensify frequency-related investigations, establish more sophisticated theoretical models for soundscape research, and actively explore the utilization of frequency components to alleviate disruptive auditory environments. Furthermore, future research should delve deeper into other acoustic indicators, such as timbre, to comprehensively comprehend the mechanisms governing soundscape preferences.

Future studies can also be expanded to include soundscapes preferred by other classical gardens of different cultures and regions in the world and to conduct a cross-cultural comparison study with the soundscapes of classical Chinese gardens in order to explore the influence of culture on soundscape preferences.

The audio samples analyzed in this study were downloaded from the BBC’s Sound Effects library, and not recorded live in classical gardens. Due to the noise reduction of the audio, the deviation of audio data from the live audio is negligible, but audio recorded in the field can be a useful supplement to the research data.

### Supplementary Information

Supplementary Information.

## Data Availability

The datasets used and/or analysed during the current study available from the corresponding author on reasonable request.
